# Supplementary material for: On-the-Fly Ring-Polymer Molecular Dynamics Calculations of the Dissociative Photodetachment Process of the Oxalate Anion
Source: Molecules. 2021 Nov 29;26(23):7250. doi: 10.3390/molecules26237250 (PMC8658898; doi:10.3390/molecules26237250)
Supplement: Supplementary file 1 [file molecules-26-07250-s001.zip › molecules-1473362-supplementary.pdf]

On-the-Fly Ring-Polymer Molecular Dynamics Calculations of the Dissociative Photodetachment Process of the Oxalate Anion

Yukinobu Takahashi [1], Yu Hashimoto [1], Kohei Saito [1] and Toshiyuki Takayanagi [1],*

[1] Department of Chemistry, Saitama University, Shimo-Okubo 255, Sakura-ku, Saitama City, Saitama 338-8570, Japan

* Correspondence: tako@mail.saitama-u.ac.jp; Tel.: +81-48-858-9113

Supplementary Material

In this Supplementary Material, we describe the accuracy of the present approximation used in the PIMD calculations, where the force acting on each bead of ring-polymer was approximately calculated from the first and second derivatives of the potential energy at the centroid position of the nuclei beads. In order to get insight into appropriate PIMD parameters (temperature and the number of beads), we have employed a different chemical system, namely, $F^{-}\cdot H_2O$ cluster, for which a semiempirical PM6 potential energy surface works reasonably well from the comparison to the ab initio results [1]. Notice that the on-the-fly calculations using the semiempirical quantum chemistry level are much faster than the on-the-fly calculations using the DFT level by a factor of about 100. The total time step is set to 40000 with a time increment of Δt = 0.25 fs and the structural distributions were then obtained.

In Figure S1, we compare the result obtained from the usual PIMD scheme (black lines), where the force acting on each bead of ring-polymer was explicitly calculated, to the result obtained from the approximated PIMD scheme using the centroid potential energy surface property (red lines). The two angular distribution functions are plotted, where their definition is also shown in Figure S1. Figure S1(a) and (b) show the results for the $F^{-}\cdot H_2O$ and $F^{-}\cdot D_2O$ clusters, respectively, with T = 200 K and 48 beads, where the convergence of this bead number was confirmed from the convergence of total energy. Although the approximate PIMD scheme gives reasonable agreement, small deviations can be seen in the broad angular distributions at 60–120 deg. This is presumably coming from the spread of the ring polymer (quantum nature) at lower temperatures. Figure S1(c) shows the result for the $F^{-}\cdot H_2O$ cluster with T = 300 K and 32 beads. It is clear that a very good agreement is obtained at

this temperature. Therefore, we have decided to perform all the PIMD (RPMD) calculations using the approximate force scheme at T = 300 K and 32 beads.

Reference

[1] Wang, Q.; Suzuki, K.; Nagashima, U.; Tachikawa, M.; Yan, S. Semiempirical investigations on the stabilization energies and ionic hydrogen-bonded structures of $F^-(H_2O)_n$ and $Cl^-(H_2O)_n$ (n = 1–4) clusters. *J. Theor. Appl. Phys*. **2013**, *7*, 7. DOI:10.1186/2251-7235-7-7

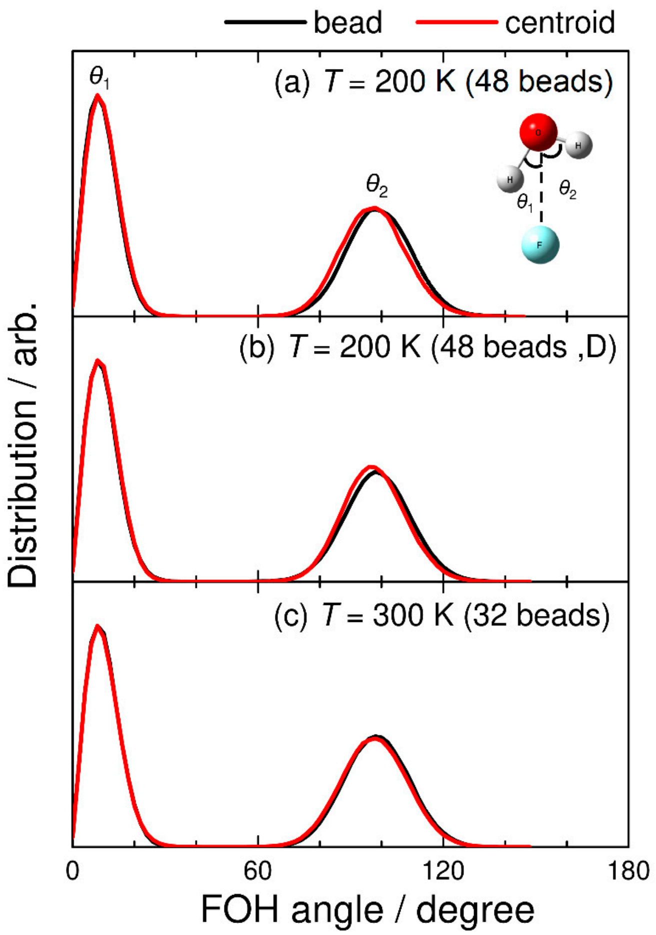

Figure S1. Angular distribution functions for the $F^-{\cdot}H_2O$ ($F^-{\cdot}D_2O$) cluster obtained from the two different PIMD schemes. The black lines show the results obtained from the usual PIMD calculations, where the force acting on each bead of ring polymer was explicitly calculated. The red lines show the results obtained from the approximate PIMD calculation using the centroid potential energy surface property.
